# L’ouverture de l’orifice interne du col prédit mieux l’issue de la maturation cervicale que le score de Bishop chez les nullipares à 41 semaines d’aménorrhée

**DOI:** 10.11604/pamj.2016.25.203.10188

**Published:** 2016-11-29

**Authors:** Mehdi Kehila, Hassine Saber Abouda, Rim Ben Hmid, Omar Touhami, Cyrine Ben Miled, Imen Godcha, Sami Mahjoub, Mohamed Badis Chanoufi

**Affiliations:** 1 Service C de Gynécologie et Obstétrique, Centre de Maternité de Tunis, Université Tunis El Manar, Tunisie

**Keywords:** Score de Bishop, échographie, orifice interne du col, maturation cervicale, Bishop’s score, ultrasound, internal cervical os, cervical ripening

## Abstract

**Introduction:**

L'objectif était d'évaluer la mesure échographique de l’ouverture de l’orifice interne du col dans la prédiction de l’issue de la maturation cervicale et la comparer au Score de Bishop.

**Méthodes:**

Nous avons mené une étude prospective sur 10 mois, entre Juillet 2012 et avril 2013 colligeant 77 femmes nullipares admises pour déclenchement du travail à un terme de 41 SA avec un Score de Bishop < 6. La mesure de l’ouverture de l’orifice interne du col a été réalisée par échographie transvaginale et le score de Bishop a été déterminé par l'examen clinique. Toutes les patientes ont eu une maturation cervicale par des prostaglandines.

**Résultats:**

La maturation cervicale était réussie chez 63 patients (81%). Le Score de Bishop et l’ouverture de l’orifice interne du col se sont révélés statistiquement associés au succès ou l’échec de la maturation cervicale. Le taux de succès de la maturation était de 100% lorsque l’ouverture de l’orifice interne du col était égale ou supérieure à 5 mm (sensibilité: 54%; spécificité: 86%). Les courbes ROC ont montré que la mesure de l’orifice interne du col était plus prédictive de l’issue de la maturation cervicale que le Score de Bishop (Aire sous la courbe respectivement 0.733 et 0.704).

**Conclusion:**

Comparée au score de Bishop, la mesure échographique de l’ouverture de l’orifice interne du col est plus prédictive du succès de la maturation cervicale chez les femmes nullipares à 41 semaines d’aménorrhée avec un col défavorable.

## Abstract (en)

**Introduction:**

The objectif was to evaluate ultrasound measurement of the opening of the internal cervical os in the prediction of cervical ripening and to compare it with Bishop’s score.

**Methods:**

We conducted a prospective study of 77 nulliparous women admitted to hospital in labor at 41w gestation, with Bishop’s Score < 6, over a 10-month period, between July 2012 and April 2013. The measurement of the opening of the internal cervical os was performed using transvaginal ultrasound and Bishop’s score was determined by clinical examination. All patients received prostaglandins for cervical ripening.

**Results:**

Cervical ripening was successful among 63 patients (81%). Bishop’s score and the opening of the internal cervical os were statistically associated with the success or failure of cervical ripening. The success rate of cervical ripening was 100% when the opening of the internal cervical os was equal to or greater than 5 mm (sensitivity: 54%, specificity: 86%). ROC curves showed that the measurement of the internal cervical os was more predictive of cervical ripening than Bishop’s score (area under the curve: 0.733 and 0.704 respectively).

**Conclusion:**

Compared to Bishop’s score, echographic measurement of the opening of the internal cervical os is more predictive of the success of cervical ripening in nulliparous women at 41 weeks of pregnancy with an unfavourable cervix.

## Introduction

Le déclenchement du travail est une des procédures de l’obstétrique moderne. Son taux a augmenté progressivement ces dernières décennies pour atteindre une incidence d’environ 20% de tous les accouchements [1]. Toutes ces inductions n’aboutissent pas à un accouchement par voie basse. Des césariennes peuvent s’avérer nécessaires soit dans un contexte d’urgence soit pour un échec du déclenchement du travail. Ceci souligne l’importance de trouver la meilleure stratégie qui permettrait d’aboutir à un taux maximal de succès de déclenchement. Une des stratégies possibles est d’essayer d’identifier la population de femmes chez qui l’induction du travail a le plus de chance de réussir. Classiquement, cette identification est basée sur le Score clinique de Bishop qui est simple et facile à établir [2]. Toutefois, plusieurs études ont montré que ce score est très subjectif avec une grande variabilité inter et intra examinateurs et un faible taux de prédiction de l’issue du déclenchement du travail surtout chez les femmes avec un Score de Bishop < 6 [3]. L’échographie cervicale constitue probablement une des avancées majeures en obstétrique ces dix dernières années. Cette évaluation échographique du col est une méthode plus objective et reproductible et a été proposée pour affiner la probabilité de succès du déclenchement [3]. Plusieurs critères échographiques ont été étudiés dans ce but dans la littérature [4]. La longueur du col est le critère échographique le plus étudié [4] avec des résultats encourageants chez les primipares [4]. La mesure de l’ouverture de l’orifice interne du col quant à elle a été peu étudiée et les résultats des études qui s’y ont intéressées sont discordants [5, 6], en partie probablement à causes des caractères hétérogènes des populations étudiées. En effet, il a été montré pour la longueur du col, l’effet délétère de l’inclusion de populations hétérogènes sur les résultats statistiques [4]. La présente étude a pour but d’évaluer l’ouverture de l’orifice interne dans la prédiction de l’issue de la maturation cervicale et le comparer au Score de Bishop dans une cohorte homogène faite de femmes nullipares avec un Score de Bishop initial défavorable et admises toutes pour un déclenchement du travail pour un terme avancé.

## Méthodes

Il s’agit d’une étude prospective réalisée sur une période de 10 mois, allant de Juillet 2012 à avril 2013 portant sur des patientes nullipares admises pour un déclenchement du travail dont l’indication était un terme avancé.

*Les critères d’inclusion dans l’étude étaient:* un âge gestationnel précis vérifié par une mesure de la longueur cranio-caudale réalisée au premier trimestre; la nulliparité; une grossesse unique; un fœtus avec activité cardiaque positive et un rythme cardiaque fœtal normal ; une présentation céphalique ; des membranes amniotiques intactes; l’absence chez la patiente d’Hypertension artérielle, de diabète ou d’autre maladie chronique connue; l’absence de contractions utérines; l’absence d’antécédent de chirurgie utérine; l’absence de contre-indication à la voie basse; un Score de Bishop < 6. Une échographie trans-abdominale était faite pour chaque patiente déterminant: la plus grande citerne du liquide amniotique, la localisation placentaire, la présentation et les biométries fœtales ainsi qu’un enregistrement cardio-tocographique.

*Les critères d’exclusion étaient :* une échographie du premier trimestre non disponible ; la présence d’une anomalie du liquide amniotique; la présence d’un retard de croissance fœtale (Biométries fœtales < 10^ème^percentile) ou d’une macrosomie (poids fœtal estimé >4000g) ; un placenta praevia ; la suspicion de disproportion foeto-pelvienne; un BMI <20 ou >29,9 Kg/m^2^.

**Figure 1:**
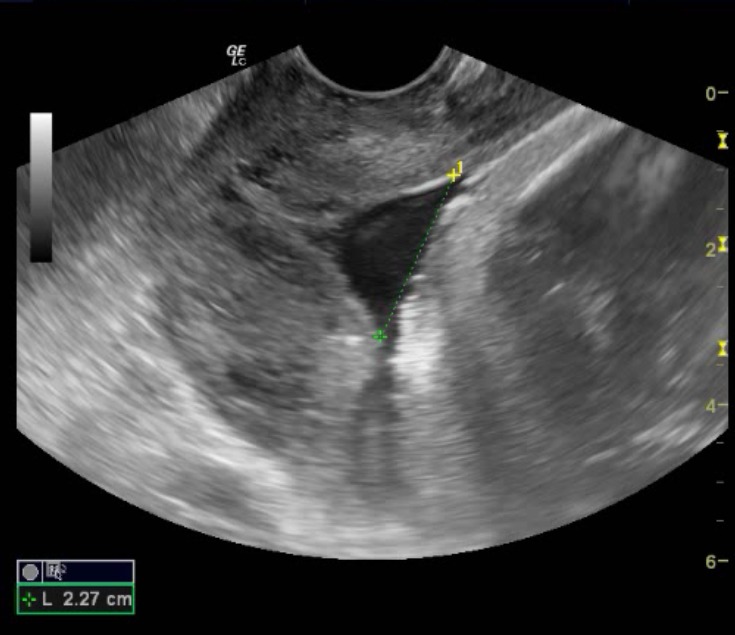
Exemple de de mesure de l’orifice interne du col

*Protocole de l’étude:* à l’admission en salle de travail et juste avant chaque administration intra vaginale d’une dose de 0,5 mg de Dinoprostone; pour chaque patiente, était réalisée une échographie trans-vaginale mesurant l’ouverture de l’orifice interne du col. Cette mesure était faite par un gynécologue obstétricien sénior ou un résident de 4^ème^ année de la spécialité entrainé à la technique. L’appareil d’échographie utilisé était un Aloka Prosound ALPHA 7 (Aloka, Tokyo, Japan) avec sonde de 6.0 MHz. La technique de mesure de l’orifice interne du col était comme suit: La sonde était placée en endovaginal visualisant l’orifice interne du col, le canal cervical et l’orifice externe du col dans un plan sagittal. Chaque examen a été pratiqué avec une vessie vide et sans pression sur le col avec la sonde pour ne pas déformer le col et fausser la mesure. Le degré d’ouverture de l’orifice interne du col était mesuré (Figure 1). Trois mesures successives étaient à chaque fois réalisées et la valeur retenue était celle mesurée sur le meilleur cliché choisi par concertation entre au moins deux séniors de l’équipe. Pour chaque patiente, le Score de Bishop a été mesuré par un obstétricien sénior. L’examinateur qui a réalisé l’échographie et celui qui a déterminé le Score de Bishop ignoraient les résultats l’un de l’autre. Le déclenchement du travail a été réalisé selon le protocole du service: Une cardiotocographie externe était d’abord réalisée pour chaque patiente pour s’assurer du bien être fœtal et de l’absence de contractions avant le début du déclenchement. Vu que le score de Bishop dans notre étude était toujours < 6, toutes nos patientes ont bénéficié d’une maturation cervicale première par des prostaglandines E2. Une première dose de 0,5 mg de Dinoprostone (Prepedil intracervical^®^ Pfizer Holding, France) était mise en intracervical. Une cardiotocographie était réalisée 30 min plus tard pour s’assurer du bien être fœtal. S’il n’y avait pas de contractions spontanées 8 à 10 heures après la mise de cette première dose avec un Score de Bishop toujours < 6, une deuxième dose de 0,5 mg de Dinoprostone était appliquée après une cardiotocographie externe confirmant l'absence de contractions et le bien-être fœtal. Une deuxième cardiotocographie était réalisée 30 min plus tard. S’il n’y avait pas de contractions spontanées 8 à 10 heures après la mise de la deuxième dose et le Score de Bishop était toujours < 6, une troisième et dernière dose de 0,5 mg de Dinoprostone était appliquée. Le succès de la maturation cervicale était défini par une atteinte d’un score de Bishop = 6 avec un maximum de 3 doses de gel de Dinoprostone. Dans ce cas une direction du travail par 5UI d’ocytocine diluées dans 500 cc de sérum physiologique et/ou une amniotomie pouvaient être réalisées selon les conditions obstétricales et les contractions utérines. L’échec de la maturation cervicale était défini par un score de Bishop toujours < 6 huit heures après la troisième dose de prostaglandines avec absence de contractions utérines spontanées. Dans ce cas, quelque soit le score de Bishop, un déclenchement du travail par 5UI d’ocytocines diluées dans 500 cc de sérum physiologique était tenté. Pour chaque patiente nous avons recueilli: l’âge, le Score de Bishop initial, le degré d’ouverture de l’orifice interne du col, le nombre de doses de gel de Prépidil utilisées, le mode d’accouchement, le poids du nouveau-né et l’Apgar à la naissance. Nous avons aussi noté toute complication maternelle ou fœtale.

*Analyse statistique:* l’analyse statistique a cherché une association entre chaque critère étudié et l’issu de la maturation cervicale ainsi que le meilleur critère pouvant la prédire. Les méthodes statistiques utilisées ont été le test t de Student, le test de Mann Whitney U. La force de l’association entre les différents paramètres étudiés et l’issue de la maturation cervicale a été mesurée par les odds ratios (OR). Des courbes ROC ont été dressés et comparées en utilisant l’aire sous la courbe afin de déterminer le paramètre le plus pertinent. L’intervalle de confiance à 95 % (IC 95 %) a été calculé. La sensibilité et la spécificité ont été calculées pour différents points de césure pour chacun des paramètres étudiés. Tous les tests statistiques étaient de formulation bilatérale, toute valeur égale ou inférieure à 0,05 définissant la signification statistique. L’analyse statistique a été réalisée avec le logiciel SPSS 19.0 (SPSS Inc II USA) pour le software Windows. Cette étude a été approuvée par le comité d’éthique local du centre de maternité de Tunis et toutes les patientes ont donné leur consentement éclairé pour la participation à l’étude.

## Résultats

**Figure 2:**
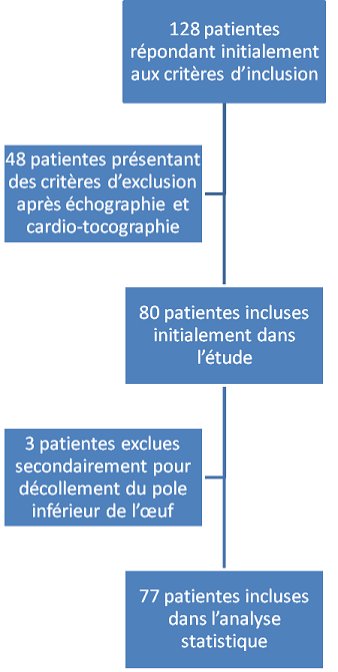
Organigramme de l’étude

Pendant la période d’étude, 2586 accouchements ont eu lieu dans le service. Trois cent trente-six déclenchements pour terme avancé ont été réalisés pendant cette même période soit 12.9% du total des accouchements. Parmi ces patientes déclenchées pour terme avancé, 299 étaient des primipares (88.9%). Cent vingt-huit patientes répondaient aux critères d’inclusion. Quarante huit parmi ces patientes ont été exclues par la suite devant la mise en évidence d’un critère d’exclusion échographique ou clinique. Quatre-vingt patientes ont enfin été incluses dans l’étude. Trois femmes ont été exclues secondairement. La raison était qu’elles aient eu, en plus de la dose de gel de Prépidil, un décollement artificiel du pole inférieur de l’œuf chose qui n’était pas admise dans le protocole de l’étude pour ne pas introduire des biais de confusion. L’analyse statistique a porté au total sur 77 patientes. La Figure 2 illustre l’organigramme de la sélection des patientes et de leur inclusion dans l’étude: La moyenne d’âge des patientes était de 28,1 ans (extrêmes 19 - 40 ans) ; L’ouverture moyenne de l’orifice interne du col était de 2.05 mm ±2.8 (extrêmes 0 - 11 mm) ; La moyenne du Score de Bishop initial était de 2,38 ±1.4 (extrêmes 0-5) ; Un succès de la maturation cervicale a été obtenu chez 63 patientes (81%) ; Aucune des patientes dans notre série n’a bénéficié d’une analgésie péridurale lors de son accouchement ; Toutes les patientes ont accouché dans les 60 heures après le début du déclenchement ; L’accouchement s’est déroulé par voie basse chez 51 femmes (66,2 %) ; Vingt-six patientes (33.8%) ont accouché par césarienne : 18 pour une dystocie de démarrage (23.4 %), 5 pour une anomalie du rythme cardiaque fœtal (RCF) (6.49%), 1 pour stagnation de la dilatation (1.31%), et 2 pour un défaut d’engagement (2.59%) ; Une extraction instrumentale a été réalisée dans 3 cas (5.88%) : Une pour un RCF pathologique lors de la phase d’expulsion et deux pour défaut d’expulsion; Trois parmi nos patientes (4.76%) ont présenté une hémorragie de la délivrance. L'hémorragie a été jugulée par un traitement médical à base de prostaglandines (Sulprostone) dans tous les cas. Les 3 patientes ont nécessité des transfusions; Tous les nouveau-nés étaient en bonne santé apparente. Aucun nouveau-né n’a présenté un Apgar < 8 à 1 min et tous avaient un Apgar = 9 à 5 min. Le poids moyen à la naissance était de 3435g (3000 - 3950g); 2 nouveau-nés ont été transférés en néonatologie pour surveillance. Ces nouveau-nés ont été surveillés pour une suspicion d’infection materno-fœtale avec une bonne évolution. Le Score de Bishop et l’ouverture de l’orifice interne du col se sont révélés statistiquement associés à l’issue de la maturation cervicale (Tableau 1).

**Tableau 1:** Ouverture moyenne de l’orifice interne du col et Bishop initial moyen en fonction de l’issue de la maturation cervicale

	Groupe « Succès de la maturation »	Groupe « Echec de la maturation »	p
Ouverture moyenne de l’orifice interne du col	3.18 ±2.9	0.21 ±0.58	<0.01
Moyenne du Bishop initial	2.54 ±1.4	1.64 ±0.7	0.02

**Figure 3:**
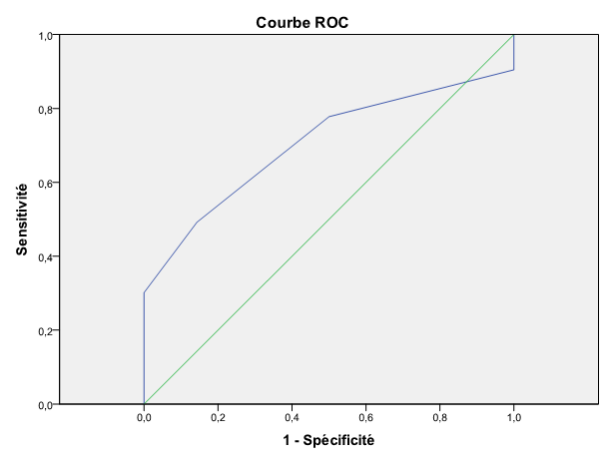
Courbe ROC pour la prédiction du succès de la maturation cervicale par le score de Bishop

**Figure 4:**
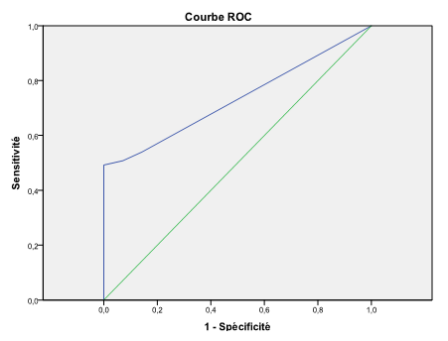
Courbe ROC pour la prédiction du succès de la maturation cervicale par la mesure échographique de l’ouverture de l’orifice interne du col

Dans le groupe « succès de la maturation cervicale », la moyenne du score de Bishop était significativement plus élevée (p=0,02) et l’ouverture de l’orifice interne était plus importante (p=0.02). Le taux de succès de la maturation était de 100% lorsque l’ouverture de l’orifice interne du col était égale ou supérieure à 5 mm (sensibilité: 54%; spécificité: 86%). Le poids fœtal à la naissance et l’âge de la patiente quant à eux n’étaient pas significativement associés à l’issue de la maturation cervicale (respectivement p=0.23 et p=0.24). En analysant les courbes ROC pour la prédiction du succès de la maturation, il en sort que la mesure de l’orifice interne du col était plus prédictive de l’issue de la maturation cervicale que le Score de Bishop (Aire sous la courbe respectivement 0.733 (IC95% = 0.62-0.85) et 0.704 (IC95%=0.58-0.83) (Figure 3 et Figure 4). Une ouverture de 1.5 mm était le meilleur « cut-off » calculé pour la prédiction du succès de la maturation cervicale (sensibilité =51%, spécificité =93%, OR=13.4, IC 95%= 2.44 - 73.8). Une ouverture de l’orifice interne du col de 1.5 mm ou plus multipliait donc les chances de succès de la maturation par 13 par rapport à un orifice interne fermé. Un Score de Bishop initial de 2 s’est avéré le meilleur « cut-off » pour le Score de Bishop dans la prédiction du succès de la maturation cervicale (sensibilité =63%, spécificité =68%, OR=3.5, IC 95%= 1.1 - 11.2).

## Discussion

Le déclenchement du travail est le processus qui consiste à donner des contractions utérines à une patiente non en travail afin de provoquer l’effacement et la dilatation du col et aboutir à la naissance. Les déclenchements pour indication médicale ou de convenance représentent près de 22.7% des accouchements [2]. Dans notre service, le déclenchement du travail a concerné 26% des accouchements pendant la période d'étude. L’indication du déclenchement peut être maternelle, fœtale ou mixte [3]. L’indication la plus fréquente dans la littérature est celle du dépassement de terme dans un but de réduire la mortalité périnatale [3]. L’étape préalable à l’induction du travail est l’évaluation du statut cervical faite classiquement par le Score de Bishop. Ce score a été défini en 1964 comme une méthode pour prédire le délai de la mise en travail des femmes multipares [7]. Secondairement, le score de Bishop a été utilisé comme méthode d’évaluation du statut cervical chez les patientes devant bénéficier d’une induction du travail toutes parités confondues et il a prouvé son intérêt clinique pour prédire le succès du déclenchement du travail [8]. Le résultat de ce score clinique déterminait, il y'a pas longtemps, à lui seul, le type de prise en charge à proposer aux patientes: maturation cervicale avant déclenchement par les ocytociques pour les patientes à score défavorable (classiquement < 6) ou déclenchement d’emblée pour les patientes à score de Bishop favorable. Mondialement utilisé depuis 1964, le score de Bishop est aujourd’hui de plus en plus souvent remis en question. En effet, les 5 critères recueillis pèsent de façon identique dans la détermination du Bishop. Or l’intuition clinique selon laquelle tous les critères n’ont pas le même poids dans l’évaluation du col est confirmée par plusieurs études, en particulier celle de Fuentes et al. [8]. Dans son travail, il montre que l’association de chaque critère avec le succès du déclenchement varie et que la dilatation cervicale est le critère majeur. Edwards [9], dans une étude plus récente a trouvé aussi les mêmes résultats. De plus, même parmi les obstétriciens expérimentés, il existe une variabilité intra et inter observateurs non négligeable dans l’appréciation du Score de Bishop. L’équipe suisse d’Irion et al. [10] a mené en 2003 une étude visant à évaluer la reproductibilité inter observateur du score de Bishop. Cinquante-six patientes ont été chacune examinées par 2 obstétriciens. La concordance parfaite entre 2 observateurs était retrouvée dans moins de 40% des cas. Tous ces arguments ont poussé les obstétriciens à essayer d’améliorer la pertinence de ce score soit en réalisant des scores simplifiés plus reproductibles soit en apportant à l’expertise clinique une expertise paraclinique moins sujette aux variations inter et intra observateurs. L’autre moyen d’évaluer facilement le statut cervical est la réalisation d’une échographie du col de l’utérus par voie vaginale. Il a par ailleurs, été démontré que 50% de la longueur cervicale n’était pas évaluable au toucher vaginal en raison de l’existence du segment supra vaginal du col. L’échographie semblant donc bien adaptée pour évaluer le col dans sa totalité. L’échographie endovaginale a d’abord été validée comme un moyen fiable et reproductible d’évaluation de la longueur cervicale [11, 12]. Goldberg [13] a montré qu’il existait une meilleure corrélation inter-observateurs dans l’évaluation de la longueur cervicale en échographie en comparaison au le toucher vaginal. Le taux de concordance dans l’évaluation de la longueur cervicale au millimètre près entre 2 observateurs étant de 35% avec le toucher vaginal contre 74% en échographie vaginale. La plupart des études qui se sont intéressées à l’échographie dans la prédiction de l’issu de l’induction du travail ont étudié le paramètre longueur du col [4]. Ce paramètre s’est révélé un bon élément prédictif de l’issu de l’induction du travail chez les patientes nullipares [4]. Toutefois, d’autres paramètres échographiques, méritent à notre sens d’être bien évalués. L’ouverture de l’orifice interne du col est un paramètre qui a été peu étudié et les résultats concernant ce paramètre sont discordants [5, 6]. Keepanasseril et al. [14] ont trouvé, dans une étude prospective à propos de 311 femmes candidates à un déclenchement du travail, que la mesure de l’ouverture de l’orifice interne du col est un bon prédicteur du succès de cette induction. Dans une autre étude rétrospective portant sur 131 patientes enceintes à terme avec grossesse monofoetale et membranes intactes candidates à une induction du travail, Neha et al. [15] a inclus l’ouverture de l’OIC dans un score se basant sur plusieurs données obtenues par échographie endo- vaginale. Les composantes de ce score étaient: la longueur cervicale, l’ouverture de l’orifice interne, la position du col et la distance orifice interne - présentation. En comparant ce score échographique au score de Bishop, l’auteur a conclu que le score échographique est meilleur dans la prédiction du succès du déclenchement du travail avec un taux de 90% d’accouchements par voie basse dans les 17h suivant le début du déclenchement pour les patientes ayant un score échographique = 4.

Deepeka N. et al. [16] dans une étude prospective incluant 150 femmes admises pour induction du travail, ont évalué des paramètres cliniques (IMC, taille des patientes, poids fœtal estimé, score de bishop) et des paramètres échographiques (position de la tête fœtale, longueur cervicale et ouverture de l’orifice interne du col). Ils ont étudié la valeur prédictive de chaque paramètre dans le succès ou l’échec du déclenchement du travail. Ils ont conclu que la moyenne de la longueur cervicale était légèrement inférieure dans le groupe succès de l’induction du travail et que l’ouverture de l’orifice interne était plus importante dans ce même groupe. Verhoeven C.J.M [5] a réalisé une revue systématique de la littérature sur les moyens d’évaluation des chances de succès d’un déclenchement du travail. Il a fait une Méta-analyse de 31 études prospectives incluant un total de 5029 femmes. Parmi ces 31 études, 8 se sont intéressées à l’ouverture de l’orifice interne du col dans la prédiction du succès du déclenchent du travail. Ces études ont conclu que l’association col long orifice interne fermé double le risque d’échec du déclenchement alors que l’association col court orifice interne ouvert, diminue le risque d’échec de 50%. Ces résultats n’étaient pas trouvés dans des études plus anciennes, notamment la méta-analyse de Hatfield al. [6] publiée en 2006 et la revue de littérature publiée par Boozarjometrie et al. [17] en 1994. Les résultats contradictoires de ces études peuvent être expliqués par l'inclusion de groupes de patientes hétérogènes de point de vu indication du déclenchement du travail [5, 6, 17] à différents âges gestationnels [5, 6, 17]. Une autre raison pour laquelle les résultats de ces études sont discordants est l’inhomogénéité des populations étudiées de point de vue parité [6, 17], puisque la réponse aux ocytociques et la dilatation cervicale chez les femmes multipares sont différentes de celles chez les femmes nullipares [4]. De plus l’antécédent d'accouchement par voie vaginale, rend le col utérin plus compliant, ce qui influe sur les résultats statistiques en cas de populations faites de femmes de parités différentes [4]. Ce biais important lié à l’étude de populations hétérogènes est amplifié par l'absence de standardisation de la méthode de déclenchement, qui comprend l’amniotomie, l'ocytocine et / ou d’autres prostaglandines [4]. Nous avons étudié dans ce travail une cohorte homogène de femmes nullipares ayant toutes un score de Bishop bas, sans aucune pathologie maternelle ou fœtale pouvant interférer avec l’issue de la maturation. Elles avaient toutes exactement le même âge gestationnel et ont toutes eu un seul et même protocole d’induction du travail. Notre étude a montré que la mesure échographique de l’orifice interne du col était corrélée à l’issue de la maturation cervicale et qu’elle était plus prédictive que le Score de Bishop dans une population de femmes avec un score de Bishop bas chez qui on a procédé à un déclenchement du travail par prostaglandine E2 à 41 SA. Un orifice interne du col ouvert à 1.5 mm ou plus multiplié par un facteur 13 les chances de succès de la maturation par rapport à un orifice interne fermé et à partir d’une ouverture de 5 mm toutes les patientes ont eu un succès de la maturation cervicale. Nous avons choisi dans notre travail d’étudier le critère « succès de la maturation » (atteinte d’un Score de Bishop favorable). En effet, ce point nous a paru particulièrement intéressant à étudier vu qu’il est bien prouvé que les femmes ayant un Bishop favorable ont plus de chance d’accouchement par voie basse après déclenchement et moins de complications maternelles et fœtales [1]. D’autant plus qu’à cette étape, toutes les patientes ont eu un traitement strictement identique. Nous reconnaissons que notre étude est limitée par le nombre pas très important des femmes incluses. Ceci était dû au choix initial des critères de sélection qui étaient très strictes. Toutefois, malgré la taille de l’échantillon, la significativité statistique a été obtenue. Grâce à cette méthodologie très stricte, parmi les séries retrouvées dans la littérature étudiant le même sujet, la notre est parmi celles qui ont contrôlé le plus les facteurs de confusion.

## Conclusion

Notre étude a montré que la mesure échographique de l´orifice interne du col et le Score de Bishop sont associées de façon significative au succès ou à l´échec de la maturation cervicale. Toutefois, la mesure de l´orifice interne du col s’est révélée plus prédictive de l‘issue de la maturation. Un orifice interne du col ouvert à 1.5 mm ou plus multiplié par un facteur 13 les chances de succès de la maturation par rapport à un orifice interne fermé et en cas d’ouverture de l’orifice interne supérieure ou égale à 5 mm, le taux de succès de la maturation cervicale était de 100%. A notre connaissance, il s’agit de la première étude s’intéressant à l’orifice interne du col dans la prédiction de l’issue de l’induction du travail faite dans une population aussi homogène de point de vu parité, terme, Bishop initial, indication de déclenchement et protocole d’induction du travail. Des études à plus larges effectifs sont, toutefois, nécessaires afin de confirmer ces résultats et pouvoir établir des scores utiles pour les décisions obstétricales.

### Etat des connaissances actuelle sur le sujet

- Le Score de Bishop est le moyen classique d’évaluation du col avant induction du travail;
- Les données sur la mesure échographique de l’orifice interne du col dans l’évaluation cervicale avant induction sont discordantes.

### Contribution de notre étude à la connaissance

- La mesure échographique de l'orifice interne du col est plus prédictive de l‘issue de la maturation que le Score de Bishop chez les nullipares à 41 SA ayant des conditions obstétricales défavorables;
- Un orifice interne du col ouvert à 1.5 mm ou plus multipliait par un facteur 13 les chances.
